# What is the right place for atypical exemplars? Commentary: The right hemisphere contribution to semantic categorization: a TMS study

**DOI:** 10.3389/fpsyg.2015.01349

**Published:** 2015-09-08

**Authors:** Maria Montefinese, Marco Ciavarro, Ettore Ambrosini

**Affiliations:** ^1^Department of Neuroscience, University of PadovaPadova, Italy; ^2^IRCCS Neuromed PozzilliPozzilli, Italy

**Keywords:** concept typicality, semantic categorization, hemispheric lateralization, rTMS, coarse activation hypothesis

Categorization helps organizing our world knowledge by classifying exemplar concepts into categories. The degree to which an exemplar is representative of its category is called *typicality* (Rosch and Mervis, 1975). Passeri et al. (2015) made the first attempt to assess its effect on semantic representations in Wernicke's area (lW) and its right homolog (rW) by interfering on their online activity with repetitive Transcranial Magnetic Stimulation (rTMS) while participants categorized typical and atypical exemplars. Responses to typical and atypical concepts were delayed, respectively, by rTMS over both areas and rW only, supporting the “coarse activation hypothesis” (Jung-Beeman, 2005), according to which semantic processing is coarser in right (RH) than left (LH) hemisphere. Despite the authors' effort to clarify hemispheric contribution to semantic categorization, their contribution is hindered by some theoretical/methodological limitations that are worth discussing.

Firstly, the authors interchangeably referred to contrasting semantic memory (SM) theories, making it difficult to conciliate their hypothesis with them. For example, in saying that “Typicality effect can be due to […] features shared or cooccuring between members of a category” (p. 319), they relied on feature-based semantic theories (e.g., McRae et al., 1997; Montefinese et al., 2014), which suppose a distributed network of featural representations in which typicality reflects featural intercorrelation (McRae et al., 1999; Montefinese et al., 2015). Contrastingly, in saying that “Typicality effect can be due to the number, proximity or binding of features” (p. 319), they relied on the spreading activation theory (Collins and Loftus, 1975), which supposes a somewhat hierarchical SM structure in which typicality -so-called criteriality- reflects the weight of links between basic-level and superordinate nodes. However, the authors left this dilemma open by seeking support in the Beeman's coarse activation hypothesis and sustaining that hemispheric differences “can be more simply traced to the different characteristics of the semantic fields and such an explanation lends itself to account for the effects regardless of any theoretical approach to the concepts organization” (p. 323). Besides, we would underline that, as far as we know, the original biological model (Jung-Beeman, 2005) was not integrated with connectionist models of the SM structure. Regrettably, thus, the authors missed the opportunity to clarify the cognitive processes implicated and did not provide the reader with adequate information to understand their (implicit) assumption that the semantic field is comparable to the SM structure and how to integrate the typicality (not to say the criteriality or feature correlation) within Beeman's theory. More importantly, Passeri and colleagues hypothesized that “processing of atypical members, whose features are more weakly or remotely correlated with other category members and that are more distant from the category, specifically involves the RH” (p. 320). However, they did not manipulate either criteriality or feature correlation but adopted concept-related typicality ratings and production frequency. Moving from the Passeri et al.'s findings, future works should try to overcome this limitation by assessing the effect of these fine-grained measures, as well as that of concept familiarity, which is known to affect several semantic tasks –including categorization tasks (Glass and Meany, 1978)– and shares variance with typicality (Montefinese et al., 2013).

Another critical point concerns the authors' hypotheses about RH role in categorization. Indeed, it is unclear how they infer that rW-rTMS should cause a response times (RTs) slowdown in atypical concepts categorization (p. 320). Rather, as suggested by the literature cited (Harpaz et al., 2009), one could make different predictions. Indeed, Harpaz et al. (2009), which also aimed to verify the Beeman's theory (Jung-Beeman, 2005), found more accurate responses and higher sensitivity to subordinate meaning blocks following RH-rTMS in a semantic decision task. Thus, even if Passeri and colleagues derived the trial timeline and rTMS protocol from Harpaz et al. (2009), they found quite different results. Nonetheless, the interpretation provided by the authors for this apparent incongruence was not fully satisfactory. Indeed, by relying on the fact that Harpaz et al. used a block design, they called into question putative “expectation effects on the task that increased the accuracy but nullified difference in RTs” (p. 324). However, Harpaz et al. found hemisphere-dependent effects on accuracy/sensitivity, with a clear double dissociation of the rTMS effect, and thus it is unclear how the rTMS could have modulated an unspecific expectation effect in a hemisphere-dependent way. Furthermore, another rTMS study showed that, in a picture-word matching task, only lW-rTMS delayed participants' response times for artifactual compared to natural categories (Fuggetta et al., 2009).

More importantly, the conclusion drawn by the authors seems to be not supported by the presented results. Indeed, given their analytical approach (i.e., the use of four-way ANOVAs), to claim that the rTMS over rW *selectively* delayed responses to atypical member names, as compared to both typical and non-member names, the crucial TMS-Condition × Typicality × Membership interaction should have been significant, but this was not the case in both by-subjects and by-items full-factorial ANOVAs. Rather, this conclusion is only supported by a TMS-Condition × Typicality interaction. However, it makes little sense to speak about typicality for non-member names, as typicality is a category-specific measure. Consequently, the authors made “a statistical error that is common in the neuroscience literature” (Nieuwenhuis et al., 2011, p. 1105; see also Gelman and Stern, 2006). Indeed, albeit the crucial TMS-Condition × Typicality × Membership interaction was non-significant, and without further justifications, Passeri et al. inappropriately performed separate ANOVAs. More importantly, in doing so they eliminated the sham (control) condition without justifications. Therefore, the Passeri et al.'s results should be taken with cautious and interpreted in light of the fact that their TMS-dependent effects were not modulated by the exemplar membership and were not related to an appropriate control condition for non-specific TMS effects.

To resume, Passeri et al.'s study does not permit disentangling among competing SM theories for the lack of control over some semantic/lexical dimensions of conceptual representation (Montefinese and Vinson, 2015) and of a clear semantic theoretical framework, impeding to fully appreciate their innovative contribution. However, it represents a valuable step toward creating a bridge between semantic and language theories that could resolve some of the vexing issues in both domains (Vinson et al., 2014), stimulating further research in this direction.

## Conflict of interest statement

The authors declare that the research was conducted in the absence of any commercial or financial relationships that could be construed as a potential conflict of interest.
